# Metabolic targets of watercress and PEITC in MCF-7 and MCF-10A cells explain differential sensitisation responses to ionising radiation

**DOI:** 10.1007/s00394-018-1789-8

**Published:** 2018-07-31

**Authors:** Natasa S. Giallourou, Ian R. Rowland, Steve D. Rothwell, Graham Packham, Daniel M. Commane, Jonathan R. Swann

**Affiliations:** 1 Department of Food and Nutritional Science, University of Reading, Reading, UK; 2 Division of Computational and Systems Medicine, Department of Surgery and Cancer, Imperial College London, London, UK; 3 Vitacress, Lower Link Farm, St Mary Bourne, Andover, Hampshire UK; 4 Cancer Research UK Centre, Faculty of Medicine, University of Southampton, Southampton, UK

**Keywords:** Breast cancer, Metabolomics, Phenethyl isothiocyanate, Radiotherapy, Watercress

## Abstract

**Purpose:**

Watercress is a rich source of phytochemicals with anticancer potential, including phenethyl isothiocyanate (PEITC). We examined the potential for watercress extracts and PEITC to increase the DNA damage caused by ionising radiation (IR) in breast cancer cells and to be protective against radiation-induced collateral damage in healthy breast cells. The metabolic events that mediate such responses were explored using metabolic profiling.

**Methods:**

^1^H nuclear magnetic resonance spectroscopy-based metabolic profiling was coupled with DNA damage-related assays (cell cycle, Comet assay, viability assays) to profile the comparative effects of watercress and PEITC in MCF-7 breast cancer cells and MCF-10A non-tumorigenic breast cells with and without exposure to IR.

**Results:**

Both the watercress extract and PEITC-modulated biosynthetic pathways of lipid and protein synthesis and resulted in changes in cellular bioenergetics. Disruptions to the redox balance occurred with both treatments in the two cell lines, characterised by shifts in the abundance of glutathione. PEITC enhanced the sensitivity of the breast cancer cells to IR increasing the effectiveness of the cancer-killing process. In contrast, watercress-protected non-tumorigenic breast cells from radiation-induced damage. These effects were driven by changes in the cellular content of the antioxidant glutathione following exposure to PEITC and other phytochemicals in watercress.

**Conclusion:**

These findings support the potential prophylactic impact of watercress during radiotherapy. Extracted compounds from watercress and PEITC differentially modulate cellular metabolism collectively enhancing the therapeutic outcomes of radiotherapy.

**Electronic supplementary material:**

The online version of this article (10.1007/s00394-018-1789-8) contains supplementary material, which is available to authorized users.

## Introduction

Breast cancer is a leading cause of cancer-related mortalities globally. Over 266,000 new breast cancer cases are projected to occur solely in the United States in 2018 accounting for over 40,000 deaths [1]. In the United Kingdom, breast cancer is the most common type of cancer in women, with nearly 53,700 new cases in 2013 and a one in eight estimated lifetime risk of diagnosis [2]. Radiotherapy is a primary treatment modality for breast cancer patients. This approach uses the fractionated delivery of high-energy X-ray beams to generate highly reactive free radicals in target tumour tissue. This causes DNA damage via lipid peroxidation or oxidative cellular respiration. Radiation-induced damage activates several signal transduction pathways whose primary role is to detect genomic injury leading to cell cycle arrest and DNA repair. In the event of substantial damage, the endogenous apoptotic machinery of the cells is triggered to inhibit further replication of the damaged DNA [3]. Radiotherapy also causes damage in healthy cells and can potentially trigger new cancer-initiating DNA mutations in local tissue. Therapeutic selectivity is, therefore, a vital issue in cancer therapy, and an ideal anticancer agent should be toxic to cancerous cells but exert minimal toxicity in healthy cells.

Watercress (*Nasturtium officinale*) belongs to the family of Brassicaceae together with broccoli, brussels sprouts and kale. Epidemiological studies suggest a link between the consumption of Brassica vegetables and a reduced risk for many types of cancers [4] including breast cancer [5, 6]. Watercress has a complex phytochemical profile characterised by high amounts of carotenoids, flavonols and glucosinolates [7] and is the main dietary source of phenethyl isothiocyanate (PEITC). Crude extracts of watercress have been shown to demonstrate strong antioxidant capacity in vitro [8, 9] and have been associated with the inhibition of the three stages of carcinogenesis: initiation, proliferation and metastasis in in vitro cancer cell models [10–12].

PEITC has been extensively shown to have direct anticancer effects in in vitro cancer models. PEITC exists in watercress as gluconasturtiin before mastication of the leaves, which exposes the parent compound to myrosinase resulting in the production of PEITC. It causes cell cycle arrest and mitochondrial damage in a wide variety of cell lines and it is a potent inducer of apoptosis [13–16]. Combined treatment of cancer cells with PEITC and established chemotherapeutic agents such as cisplatin and doxorubicin potentiates their cancer-killing properties [17, 18]. These findings support the potential of PEITC to be used as an adjuvant treatment during radiotherapy in cancer patients. Due to its highly electrophilic nature PEITC reacts with cellular thiols such as glutathione, the major intracellular antioxidant, depleting the cells of their antioxidant content and impacting cell survival [13, 19, 20]. As radiotherapy works primarily by inducing DNA damage through the formation of free radicals, the ability of PEITC to deplete the radical scavenger glutathione is likely to contribute to its radio-sensitising properties.

Metabolic regulation is a determining factor of the cell growth machinery and cancer cells have adapted to several oncogenic signals to modify their metabolic phenotype to support their needs for growth, survival and malignant transformation [21]. To our knowledge, limited work has been performed on the effects of isothiocyanates or of crude watercress extract on cancer cell energetics and metabolism. In this work, a metabolic profiling approach has been used to study the biochemical response of MCF-7 breast cancer cells and immortalised but non-tumorigenic MCF-10A cells to increasing doses of watercress extract (lacking PEITC) and PEITC alone. The impact of PEITC or watercress extract on the biomolecular events exerted by X-ray irradiation exposure was then investigated in these breast cells. Combining high-resolution metabolic phenotyping with measures of cell viability and DNA damage enables the radio-sensitising or radio-protective potential of watercress and its components to be evaluated.

## Materials and methods

### Cell culture

The MCF-7 human breast adenocarcinoma cell line was purchased from the American Type Culture Collection (ATCC) (LGC standards, Middlesex, UK). Cells were cultured in Dulbecco’s Modified Eagle’s Medium (DMEM) supplemented with 10% (v/v) foetal bovine serum, 2 mM glutamine, 50 U/ml penicillin and 50 U/ml streptomycin and 1% non-essential amino acids. The MCF-10A, non-tumorigenic breast epithelial cell line was purchased from ATCC (LGC standards, Middlesex, UK). Cells were maintained in Ham’s F12:DMEM (1:1), 20 ng/ml epidermal growth factor (EGF) (PeproTech, London, UK), 0.1 µg/ml cholera toxin, 10 µg/ml insulin, 500 ng/ml hydrocortisone, 5% horse serum and 50 U/ml penicillin and 50 U/ml streptomycin.

### Treatments and irradiation

For the watercress extract, fresh watercress samples were obtained directly from Vitacress Salads Ltd. (Andover, UK). Samples were snap frozen in liquid nitrogen and stored at − 80 °C. 2 g of leaf and 2 g of stem were weighed and placed in a 20 ml syringe that had the plunger removed and a circular 25 mm glass microfiber filter placed at the bottom. The syringe was then placed inside a 50 ml centrifuge tube without the lid and centrifuged at 1500*g* for 30 min to collect the extract. This crude watercress extract was then filtered through a 0.22 µm filter and used in the cultures. Phytochemical characterisation of the watercress extract has previously been published [7]. As PEITC is produced after consumption following exposure to myrosinase, it is absent from the watercress extract. To examine the metabolic effects of PEITC, 30 mM of PEITC was made up in DMSO fresh on the day of use. MCF-7 and MCF-10A cells were seeded at 1 × 10^5^ cells per well into six well plates and treated at 80% confluence. Cells were exposed to the watercress extract at 6.25, 12.5, 25 and 50 µl/ml and PEITC at 5, 10, 20, 30 µM for 24 h. Following the watercress extract/PEITC treatment period, the cells were exposed to 5 Gy X-ray radiation using an orthovoltage X-ray unit (Gulmay Medical D3225, Xstrahl, UK). The irradiator was at a stable distance from the cell culture plates and the irradiator field was approximately 20 × 20 cm. The cell culture plates were placed in the centre of the irradiation field. Following radiation treatment cells were returned in the incubator and were allowed to rest for 1 h. The cells were then collected and used in the experiments.

### Cell proliferation and viability

#### DAPI staining

For the determination of cell proliferation MCF-7 and MCF-10A cells were seeded in 96-well microplates at 5 × 10^3^ cells per well and incubated at 37 °C with 5% CO_2_ and 95% humidity for 24 h. Cells were exposed to the respective treatments and then permeabilized with 100 µl of ice-cold methanol for 5 min at room temperature. Methanol was removed and the plates were allowed to air-dry for 15 min in a hood, followed by addition of 100 µl of DAPI in PBS (70 µl of 30 mM DAPI stock solution in 10.43 ml of PBS). Cells were incubated in the dark for 30 min at 37 °C and absorption was measured using GENios microplate reader (TECAN Group Ltd., Mannedorf, Switzerland) with absorbance at 340 nm and emission at 465 nm. The experiment was performed in triplicate with three technical replicates per experiment.

#### MTT assay

Cell viability was assessed using the MTT [3-(4,5-dimethylthiazol-2-yl)-2,5-diphenyl tetrazolium bromide]-based in vitro toxicology assay kit (Sigma–Aldrich, Dorset, UK) according to the manufacturer’s instructions, in the case of the irradiation experiments. The experiment was performed in triplicate with three technical replicates per experiment.

### ^1^H NMR spectroscopy-based metabolic phenotyping

The metabolic profiles of MCF-7 and MCF-10A cells were measured using ^1^H NMR spectroscopy. Cells were seeded and treated as described above. Media was transferred into Eppendorf tubes and cells on the surface of the plate were washed twice using 1 ml of cold (4 °C) PBS and were quenched using 1 ml of ice-cold methanol (maintained on dry ice). Cells were allowed to lyze for 2 min and were detached from the plate using a cell scraper and transferred into an Eppendorf tube. Methanol quenching was repeated to maximize metabolite recovery. A vacuum concentrator (SpeedVac) was used to dry down the cell suspensions before reconstitution in 80 µl of phosphate buffer (pH 7.4) in 100% deuterium oxide containing 1 mM of the internal standard, 3-(trimethylsilyl)-[2,2,3,3,^−2^H_4_]-propionic acid (TSP).

For every sample, a standard one-dimensional NMR spectrum was acquired using a 600 MHz Bruker NMR spectrometer, with water peak suppression using a standard pulse sequence [recycle delay (RD)-90°-*t*_1_–90°-*t*_m_-90°-acquire free induction decay (FID)]. For each spectrum, 256 scans and 8 dummy scans were obtained, collected in 64K data points with a spectral width of 12.001 ppm. ^1^H NMR spectra were manually corrected for phase and baseline distortions and referenced to the TSP singlet at *δ* 0.0. Spectra were digitised using in-house Matlab (version R2016a, The Mathworks, Inc.; Natwick, MA) scripts. Metabolites were identified using an in-house database of standards. Multivariate modelling, including principal component analysis (PCA) and orthogonal projections to latent structure discriminant analysis (OPLS-DA), were performed using in-house scripts in Matlab.

### Cell cycle

Watercress extract and PEITC-treated MCF-7 and MCF-10A cells were collected by centrifugation and were then fixed in 70% (v/v) fresh ice-cold ethanol. The samples were then stored at − 20 °C until analysis. For the analysis, samples were centrifuged and the cell pellets were resuspended in 200 µl of PBS and 25 µl of 1 mg/ml RNAse added to the suspensions. The samples were incubated at 37 °C for 30 min and 2.5 µl of 400 µg/ml of propidium iodide dye were added to the cells which were then incubated for a further 30 min at room temperature under dark conditions. The final volume of the cell suspensions was adjusted to 600 µl with PBS. Cellular DNA content of 15,000 cells was quantified via flow cytometry. The flow cytometry analysis was performed using the FL2 channel on a BD Accuri™ C6 flow cytometer (Germany). Data analysis was facilitated using the Flow Jo software (version 7.6, Tree star Inc, Oregon, USA). Cell cycle progression was evaluated accounting for the percentage of all cells in each of the phases (G1, S, G2/M).

### Comet assay

The Comet assay was used for the measurement of DNA strand breaks in single cells. Treated cell suspensions were adjusted to a concentration of 1 × 10^6^ cells/ml and 50 µl were resuspended in 85 µl of warm low-melting point agarose (0.85% w/v) and applied on Trevigen Comet slides. The slides were allowed to solidify at 4 °C for 10 min. The slides were then transferred into a staining jar, lysis buffer was added (2.5 M NaCl, 0.1 M EDTA, 0.01 M Tris and 1% (v/v) Triton X—added just prior to use—pH 10), and the cells were lysed for 1 h at 4 °C. Following lysis of the cells, the slides were placed in a horizontal electrophoresis tank and incubated for 20 min in alkaline buffer (0.3 M NaOH, 1 mM EDTA—pH 13) at 4 °C in dark conditions. Subsequent to DNA unwinding, electrophoresis was carried out at 26 V, 300 mA for 20 min at 4 °C. Immediately after electrophoresis, the slides were washed in neutralising buffer (0.4 M Tris—pH 7.5) three times for 5 min. Slides were stained with ethidium bromide (20 µl/ml) and DNA migration from the nucleus was visualised with a fluorescence microscope (Olympus BX51). The computer-based image analysis software, Komet 4.0 (Andor Technology, South Windsor, CT) was used to calculate % tail DNA, the proportion of DNA migrated from the head to the tail of the comet. The mean value from 75 randomly scored cells was taken as an index of damage for each replicate well.

## Results

### Cell proliferation and viability

**Fig. 1:**
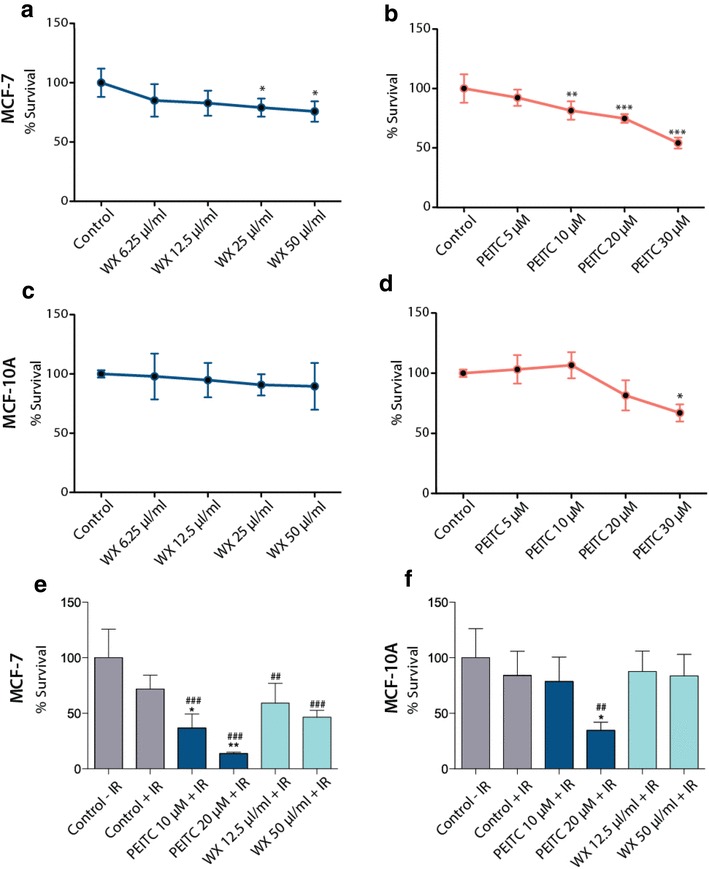
Cytotoxicity of the crude watercress extract and PEITC in MCF-7 (**a, b**) and MCF-10A (**c, d**) cells. Effect of PEITC and watercress extract (WX) pre-treatment (24 h) combined with 5 Gy of IR on MCF-7 (**e**) and MCF-10A (**f**) cell proliferation. Data presented as mean ± SEM percentage cell survival. Statistically significant differences between control and treated cells are indicated *p < 0.05, **p < 0.01, ***p < 0.001 (**a**–**d**), ^##^*p* < 0.01, ^###^*p* < 0.01 for comparisons to ‘Control−IR’ *p < 0.05, **p < 0.01 for comparisons to ‘Control + IR’ (**e**–**f**) after one-way ANOVA followed by Dunnett’s multiple comparison test. Data shown represent the average of three independent experiments with three replicates per sample. *WX* watercress

Impact on cell proliferation of increasing doses of the crude watercress extract and PEITC was assessed in MCF-7 (wild type p53) and MCF-10A cells. The dose response curves for proliferation as assessed by DAPI staining are presented in Fig. 1a–d. Watercress extract treatment did not alter MCF-10A proliferation but caused a 20% and 25% decrease in proliferation in MCF-7 cells treated with 25 and 50 µl/ml of the extract, respectively. PEITC caused a significant decrease in cell proliferation in MCF-7 cells reaching up to 46% inhibition at the highest PEITC dose (30 µM). Treatment of the MCF-10A cells with PEITC showed evidence of inhibition of proliferation only at the highest dose (30 µΜ).

The combination of PEITC or watercress treatment and IR on the overall metabolic activity of the cells was assessed using the MTT assay as a proxy of viable, metabolically active cells. Although there was a trend for IR to decrease mean reducing activity in both cell lines, this was not statistically significant (*p* > 0.05). Significant decreases in the metabolic activity of both cell lines were observed with PEITC pre-treatment prior to IR (Fig. 1e, f). At 20 µM, PEITC enhanced the effects of IR on cell viability reducing cell viability by 86% in MCF-7 (*p* < 0.01) and 69% in MCF-10A (*p* < 0.05) relative to IR alone. 10 µM of PEITC did not affect MCF-10A cell viability.

Exposure to the watercress extract prior to IR did not have an impact on cell viability in MFC-10A cells compared to irradiated cells. Combined exposure to watercress and IR resulted in significant decrease in cell viability of MCF-7 cells in both 12.5 µl/ml (41%) and 50 µl/ml (53%) compared to the non-irradiated controls (*p* < 0.01).

### Cell cycle

**Fig. 2:**
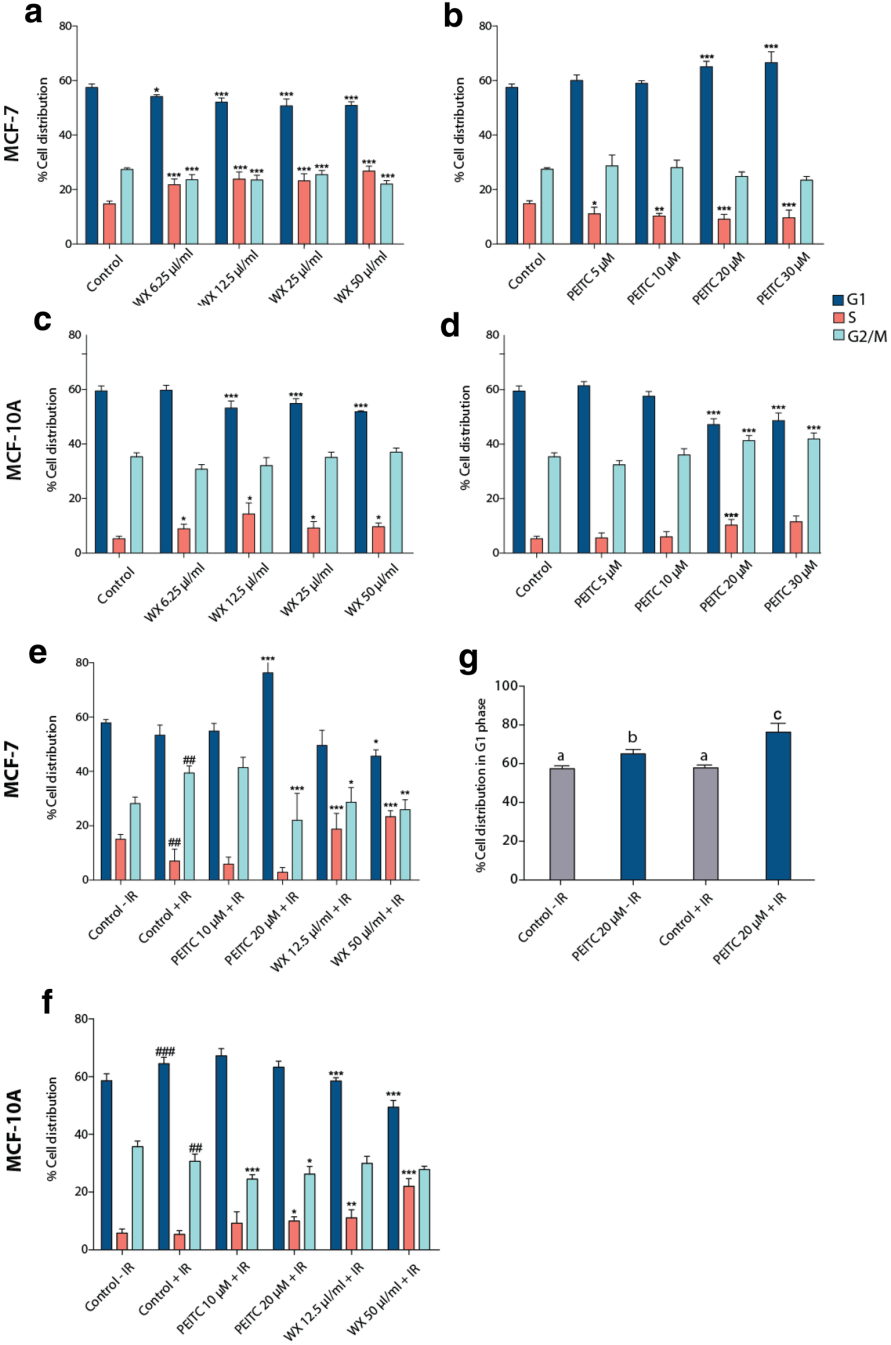
Cell cycle analysis of MCF-7 (**a, b**) and MCF-10A (**c, d**) exposed to a range of crude watercress extracts (0–50 µl/ml) and PEITC (0–30 µΜ) for 24 h, MCF-7 (**e**) and MCF-10A (**f**) cells exposed to 5 Gy of IR following a 24 h pre-treatment with PEITC or crude watercress extract (**g**). % Distribution of MCF-7 cells in G1 upon treatment with PEITC or IR. Different letters suggest statistical significance (*p* < 0.05). Data presented as mean ± SEM percentage cell distribution. Statistically significant differences between control and treated cells are indicated *p < 0.05, **p < 0.01, ***p < 0.001 (**a**–**d**), ^##^*p* < 0.01, ^###^*p* < 0.01 for comparisons to ‘Control − IR’ *p < 0.05, **p < 0.01 for comparisons to ‘Control + IR’ (**e**–**f**) after one-way ANOVA followed by Dunnett’s multiple comparison test. Data shown represent the average of three independent experiments with three replicates per sample. *WX* watercress

At the basal level, untreated MCF-7 cells had 10% greater cell distribution in the S phase and 8% in the G2 phase compared with untreated MCF-10A cells. In MCF-7 cells (Fig. 2a), the watercress extract (50 µl/ml) caused a significant 11% reduction in the G1 phase and a parallel increase in the proportion of the cells in the S phase. PEITC induced a cell cycle arrest at the G1 phase only at the highest doses (13% and 16% increase respectively) with a concomitant decrease in the proportion of cells in the S phase (39 and 34% decrease at 20 µM and 30 µM respectively) compared to non-treated cells (Fig. 2b). A similar effect of the watercress extract was observed with the MCF-10A cells with an 8% reduction of cells in the G1 phase and a 4% increase of cells in the S phase (50 µl/ml) (Fig. 2c). In contrast, PEITC did not induce a cell cycle arrest at the G1 stage as observed in the MCF-7 cells. PEITC caused a significant increase in the percentage of MFC-10A cells in the S (20 µM: 21%, 30 µM: 18% increase) and G2 phase (20 µM: 94%, 30 µM: 118% increase) and a concomitant decrease of the cells in the G1 phase only at the two highest doses (Fig. 2d).

MCF-7 cells responded to IR treatment with an accumulation of cells in G2 arrest coupled to a decrease in the number of cells in S phase. In contrast the MCF-10A cells responded to IR by increasing the proportion of cells in the G1 phase of the cell cycle (~ 6%) (Fig. 2e, f) coupled to an 11% reduction in the percentage of cells in G2.

Pre-treatment of MCF-7 and MCF-10A cells with the watercress extract or with PEITC differentially sensitised cells to a subsequent dose of 5 Gy IR, (Fig. 2e, f). In the MCF-7 cells, pre-treatment with PEITC (20 µM) led to a further reduction in the number of irradiated cells in S phase, and an accumulation of irradiated cells in G1 cell cycle arrest with a reduction in the proportion of cells in G2 relative to non-pretreated irradiated controls. The same dose of PEITC alone caused a 7.6% increase in the proportion of the cells in G1 phase but when combined with IR the proportion increased to 18.4% (Fig. 2g). In the MCF-10A cells, PEITC caused a minor decrease in the proportion of the cells on the G2 phase coupled to an increase in the percentage of the cells in the S phase.

In contrast to PEITC, watercress increased the percentage of IR cells in S phase in both cell lines. In the MCF-7 cells, this was coupled to a decrease in the proportion of cells in G2, whereas in the MCF-10A cells the proportion of cells in G1. These observations were not seen in non-irradiated watercress-treated cells.

### Comet assay

**Fig. 3:**
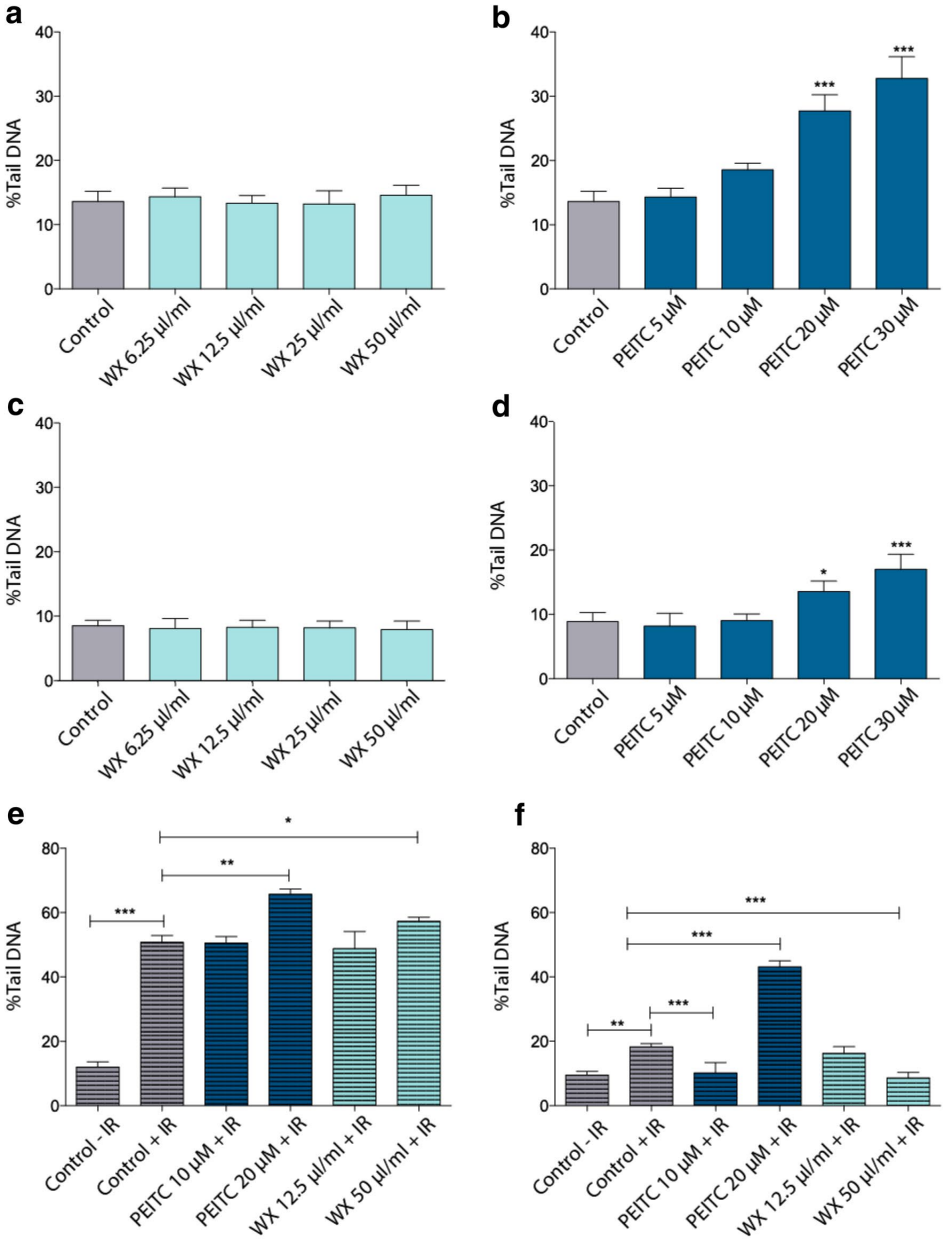
Genotoxic effects of the crude watercress extract and PEITC on MCF-7 (**a, b**) and MCF-10A (**c, d**) cells after a 24-h incubation. DNA damage levels in MCF-7 (**e**) and MCF-10A (**f**) cells exposed to 5 Gy of IR following 24 h pre-treatment with PEITC or crude watercress extract. Data presented as mean ± SEM percentage %tail DNA. Statistically significant differences between control and treated cells are indicated *p < 0.05, **p < 0.01, ***p < 0.001, after one-way ANOVA followed by Dunnett’s multiple comparison test. Data shown represent the average of three independent experiments with three replicates per sample. *WX* watercress

To further assess if the observed cell cycle arrest was a response to DNA damage, DNA lesions were quantified using the Comet assay. MCF-7 and MCF-10A cells were treated with PEITC or the watercress extract for 24 h and exposed to 5 Gy of IR or control treatment. A mild difference in basal DNA damage was observed between MCF-10A cells (8.8 ± 1.4%) and MCF-7 cells (13.6 ± 1.6%) (Fig. 3a–c). Crude watercress extract did not induce any significant genotoxic effects in either cell line at any of the concentrations tested (Fig. 3a–c). In contrast, PEITC was genotoxic in both cell lines at 20 µM with significantly increased % tail DNA. At 20 µM, PEITC caused 14.1% additional damage in MCF-7 cells and 4.7% additional damage in MCF-10A cells (Fig. 3b–d).

IR treatment induced a 39% increase in tail DNA in MCF-7 cells and pre-treatment with 20 µM of PEITC significantly increased the damage by a further 15% resulting in a final 66% tail DNA content (Fig. 3e). Pre-exposure of the MCF-7 cells to 50 µl/ml of the watercress extract also increased DNA damage by an additional 7% compared with the irradiated only cells.

MCF-10A cells exhibited sensitivity to IR (11% increase in tail DNA) but to a lesser extent than the cancerous MCF-7 cells (39% tail DNA). A 24 h pre-treatment with 10 µM of PEITC and 50 µl/ml of watercress extract reduced the Comet tail from 19.37% in the irradiated cells to 13.88 and 10.5%, respectively. The highest dose of PEITC combined with 5 Gy of IR was genotoxic to the non-tumorigenic cells resulting in a final 44% tail DNA (Fig. 3f).

### Comparative metabolic profiling of MCF-7 and MCF-10A cells

Metabolic profiles were acquired from the hydrophilic methanol extracts of MCF-7 and MCF-10A cells using ^1^H NMR spectroscopy. An orthogonal projection to latent structures discriminant analysis (OPLS-DA) model was built for a pair-wise comparison between the two cell lines. An OPLS-DA model with strong predictive ability (*Q*^2^*Y* = 0.56) was obtained and validated by permutation testing (1000 permutations; *p* = 0.001). The correlation coefficients plot from this model is presented in Fig. S1a. MCF-7 cells contained greater amounts of glutathione, as well as the amino acids alanine, glutamine, methionine, threonine and glycine, lactate, phosphocholine and glycerophosphocholine. MCF-10A cells contained greater amounts of leucine, valine, acetate, glutamate, aspartate, creatine, creatine phosphate, choline, glucose taurine, myo-inositol, the aromatic amino acids phenylalanine and tyrosine compared to the MCF-7.

### Comparative metabolic impact of IR in MCF-7 and MCF-10A cells

**Fig. 4:**
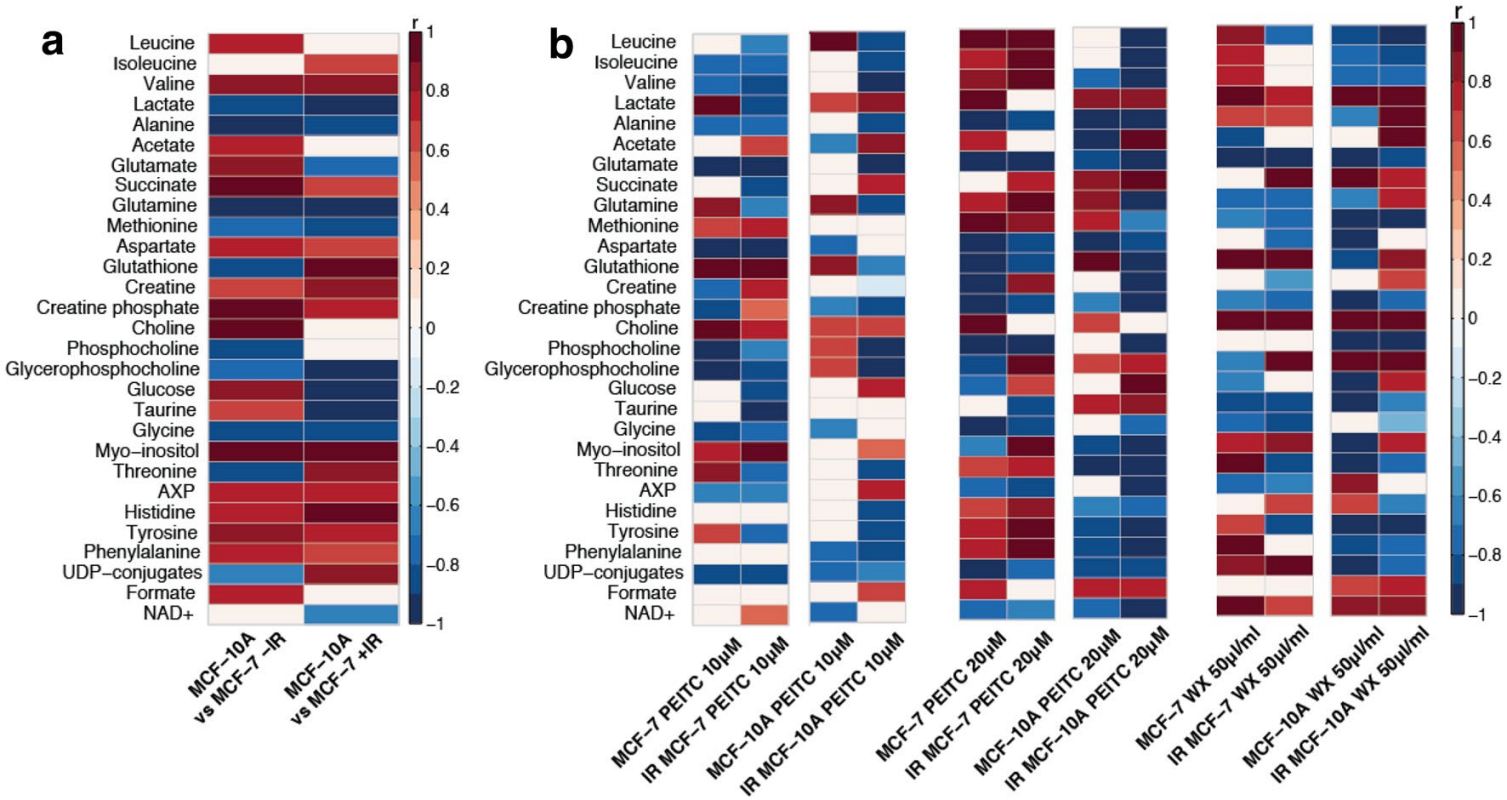
**a** Summary of the significant metabolic alterations identified from the OPLS-DA models comparing metabolic profiles of MCF-7 and MCF-10A cells with (+ IR) and without (− IR) radiation exposure (*n* = 5–6). Colours indicate the correlation coefficient (r) extracted from the OPLS-DA model. Red indicates metabolites that are present in higher amounts in MCF-10A cells and blue indicates metabolites that are present in lower amounts in MCF-10A cells compared to MCF-7 cells. **b** Summary of the metabolites associated with the OPLS-DA models given by the correlation coefficient (*r*) with the response variable, in this case PEITC or watercress (WX) treatment and IR (*n* = 5–6) in comparison to non-irradiated control or irradiated control cells. The red colour indicates metabolites that are positively correlated with the respective treatment (PEITC or WX) and blue colour indicates a negative correlation between metabolites and treatment

Comparing the metabolic profiles of irradiated MCF-7 and MCF-10A cells returned a significant OPLS-DA model (*Q*^2^*Ŷ* = 0.90, *p* = 0.001) (Fig. S1b). Following irradiation, the metabolic differences between the cell types were observed with a greater abundance of lactate, alanine, glutamine, and glycine in the irradiated MCF-7 cells compared to irradiated MCF-10A cells. However, analysis of metabolic associations (correlation coefficients) summarised in Fig. 4a identified a substantial shift in glutathione between the two cell lines. At baseline, MCF-7 cells contained higher amounts of glutathione compared to MCF-10A cells but post IR, this was reversed with MCF-10A cells containing higher glutathione. In addition, MCF-10A cells had lower amounts of phosphocholine pre-IR compared to MCF-7 cells, but this difference was no longer significant post IR suggesting a higher phosphocholine utilization rate by the non-tumorigenic cell line upon IR exposure.

### Metabolic effects of watercress and PEITC in MCF-7 and MCF-10A cells

**Fig. 5:**
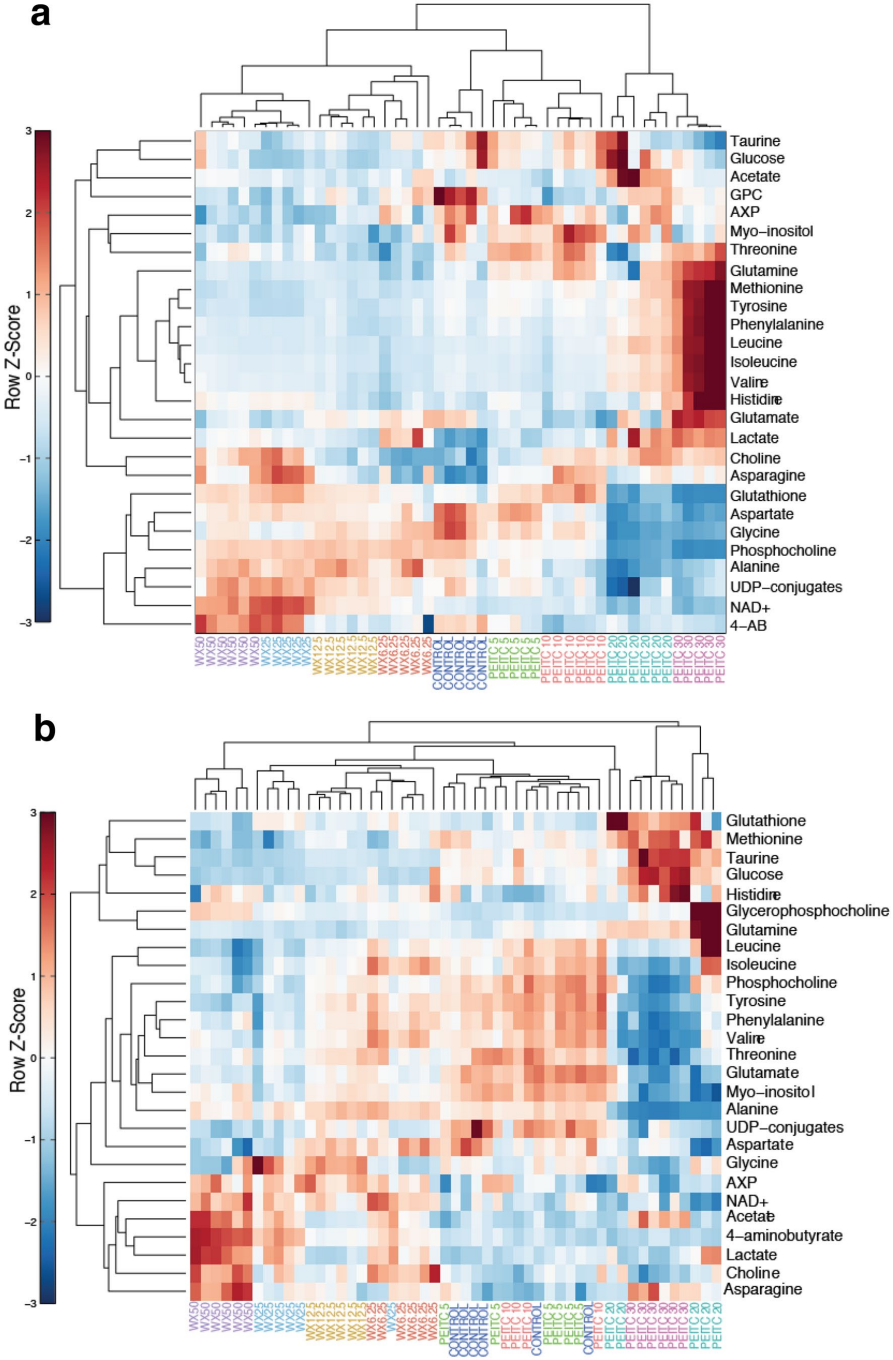
Unsupervised hierarchical clustering heat-map of metabolites from MCF-7 (**a**) and MCF-10A (**b**) cells treated with watercress extract or PEITC at increasing concentrations. Each row represents a metabolite and each column represents a sample. The row *Z* score (scaled expression value) of each metabolite is plotted in red–blue colour. The red colour indicates metabolites that are high in abundance and blue indicates metabolites low in abundance

Unsupervised hierarchical clustering of the metabolite peak integrals was performed to elucidate the metabolic shifts induced in the two cell lines following 24-h treatment with increasing concentrations of the watercress extract or PEITC. The results are summarized in Fig. 5a, b. Clear metabolic variation can be seen across the treatments and doses in the MCF-7 cells. A different biochemical response to the treatments was observed in the MCF-10A cells. At the low doses (5, 10 µΜ), PEITC induced no observable metabolic alterations in the MCF-10A cells. In the MCF-7 cells, watercress and PEITC treatments induced contrasting metabolic responses. Glutathione, aspartate, glycine, phosphocholine and alanine were significantly reduced in the MCF-7 cells treated with higher doses of PEITC but were increased in the watercress extract-treated cells. The amino acids, threonine, glutamine, methionine, tyrosine, phenylalanine, leucine, isoleucine, valine and histidine, were all elevated in the PEITC-treated MCF-7 cells and reduced in the watercress-treated groups. A more uniform response to watercress and PEITC exposure was observed in the MCF-7 cells compared to the MCF-10A cells.

### Metabolic perturbations induced by IR combined with PEITC or watercress extract pre-treatment

**Fig. 6:**
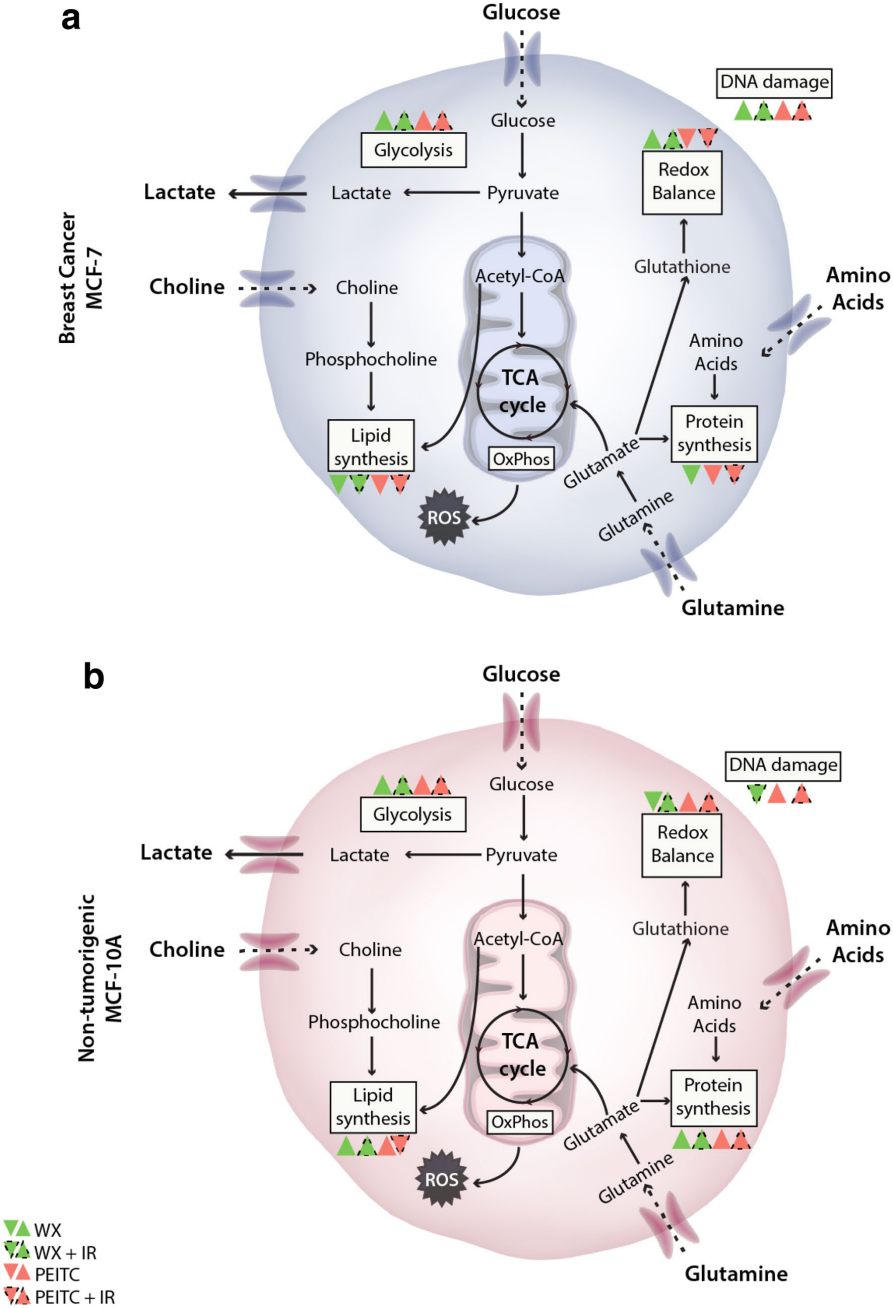
Summary of metabolic perturbations induced in MCF-7 (**a**) and MCF-10A (**b**) cells treated with the watercress extract or PEITC and IR. Green triangles indicate WX treatment and red triangles indicate treatment with PEITC. Dotted-lined triangles indicated IR exposure following WX or PEITC treatment

Figure 4b summarizes the metabolic response of each cell type to watercress or PEITC treatment and exposure to IR. Changes in cellular glutathione content were the most striking metabolic changes. A biphasic response of MCF-7 to PEITC doses with regards to glutathione was observed. Low PEITC exposure (10 µM) increased the glutathione content of MCF-7 cells but at the high PEITC dose (20 µΜ) glutathione was depleted. This effect of PEITC in MCF-7 cells persisted after exposure to IR. In MCF-10A cells, PEITC increased glutathione at both low and high doses, but glutathione was significantly decreased after exposure of the PEITC pre-treated cells to IR. Crude watercress extract treatment alone, caused an increase in glutathione in MCF-7 cells and this effect persisted following IR. In contrast, a reduction in glutathione was seen in MCF-10A cells following watercress treatment but was elevated when IR followed the watercress treatment. Most of the metabolic changes induced by the watercress extract and PEITC treatment persisted after exposure to IR in both cell types. It should also be noted, that the metabolic signature of the MCF-10A cells pre-treated with the high PEITC dose (20 µM) followed by IR exposure, is suggestive of a metabolic shut-down in these cells. This is characterised by the depletion of most of the metabolites. The metabolic pathways influenced by IR combined with PEITC or the watercress treatment in each of the cell lines are depicted in Fig. 6.

## Discussion

This study combined in vitro experiments with high-resolution metabolic phenotyping to show that watercress and a derivative of watercress, PEITC, can enhance the outcomes of radiotherapy *via* different molecular mechanisms. The protective effect of watercress in healthy cells observed here is unlikely to be a result of PEITC or any other isothiocyanate since they are not present in the extract, as a result of the high volatility of these compounds as well as the snap freezing of the plant material, which inactivates the myrosinase enzyme that converts the glucosinolate precursor to the reactive isothiocyanate. As such, PEITC was absent from the watercress extract and its effects were studied independently. Both the extract and PEITC were found to modulate the metabolome of healthy and cancerous breast cells, influencing phospholipid and amino acid metabolism as well as modulating the energy and antioxidant status of these cells. These perturbations occurred alongside cell cycle arrest, DNA damage and compromised cell viability in cancerous cells. Subsequently, it was shown that through its ability to deplete glutathione, PEITC sensitizes breast cancer cells to radiation-induced damage, whereas the watercress extract protected healthy cells from IR toxicity by increasing intracellular glutathione.

Baseline metabolic variation was clear between the cancerous MCF-7 cell line and the non-transformed MCF-10A cell line. Greater lactate content in the cancerous cells reflects a higher rate of glycolysis in these cells for energy generation, consistent with the Warburg effect. MCF-7 cells also contained greater amounts of glutamine, an amino acid required for protein and nucleotide synthesis. Similarly, phosphocholine was present in higher amounts in the MCF-7 cells, relative to the non-transformed cells, and likely relates to the higher proliferation rate in these cells and the increased synthesis of new cellular membranes, reflected in the higher proportion of cells in the S phase of the cell cycle compared with the MCF-10A cells. The cancerous cells also contained greater amounts of glutathione compared to the healthy cells. Glutathione serves as an intracellular antioxidant and its increased abundance facilitates the maintenance of appropriate cellular redox status by keeping the amount of ROS at a level that enables cell proliferation and successful progression through survival pathways as a result of post-translational modifications [22, 23]. Conversely, if the levels of ROS become extremely high this causes detrimental oxidative stress via macromolecular damage, senescence [24] and loss of mitochondrial membrane potential leading to apoptosis [25]; a collection of events that can have lethal effects on cells. To counter the outcomes of oxidative stress, cancer cells increase their antioxidant content (mainly glutathione), limiting the accumulation of ROS at excessively high levels preventing irreparable damage [26]. Naturally, proliferating cells acquire oncogenic mutations, which favour anomalous energy metabolism and protein translation leading to aberrantly increased ROS [27]. Through further mutations and adjustments, cancer cells tightly orchestrate the cycling of ROS and antioxidant production in a manner that permits cell survival and maintenance of ROS at moderate quantities.

Radiotherapy is an important treatment modality in breast cancer. This approach generates reactive free radicals, which damage DNA, ultimately resulting in cell death. Cancer cells posses several aberrant signalling pathways that can result in drug resistance or failure of therapeutic outcomes. Current research suggests that combination therapy can kill cancer cells more efficiently via diverse mechanisms simultaneously [28]. Isothiocyanates, such as PEITC, have a range of cellular targets for cancer-related outcomes, including cell cycle arrest, apoptosis, and anti-angiogenic effects [28]. As such, PEITC and its dietary source watercress are an attractive option for combinatorial therapeutic methods.

In the cancerous MCF-7 cells, IR caused DNA damage that resulted in G2 cell cycle arrest but there was no significant impact on cell survival suggesting a potential resistance of these cells to IR killing. Our results are consistent with those of Jänicke et al. [29] who observed the same cell cycle arrest and failure of IR to activate the mitochondrial intrinsic apoptosis pathway. However, pre-treatment with PEITC resulted in significant G1 arrest parallel to increased DNA damage and a significant loss of cell viability. This is likely mediated by the ability of PEITC to induce p53 activity in MCF-7 cells [30], which is a potent regulator of G1 cell cycle arrest. PEITC can also induce apoptosis from the mitochondria in breast cancer cells by caspase activation, as well as changes in the Bac/Bcl-2 ratio following the release of cytochrome *c*, all significant elements of the intrinsic apoptotic pathway [31]. In contrast, lower DNA damage was seen in the MCF-10A cells following PEITC and IR exposure.

Differences in glutathione content between MCF-7 and MCF-10A cells may contribute to the variation seen in response to PEITC and IR. Consistent with other studies, treatment of MCF-7 cells with IR-depleted intracellular glutathione [32–34]. In contrast, MCF-10A cells responded to IR induced stress by increasing their glutathione content. IR generates ROS, which are quenched in part through the glutathione response reducing the potential of ROS to exert oxidative DNA damage. Elevations in intracellular glutathione in MCF-10A cells can be considered part of a protective response by upregulating the metabolic antioxidant capacity of these cells. This may explain their ability to better recover from IR-induced damage compared to MCF-7 cells and may explain the lower DNA damage observed in the healthy cells in this study.

PEITC appears to induce a biphasic response in the glutathione abundance of MCF-7 cells, with increased concentrations at low doses and depletion at the two higher doses. The ability of isothiocyanates to act as both pro-oxidants and indirect antioxidants may explain these dose-dependent fluctuations. Prolonged exposure to low isothiocyanate concentrations can induce phase II enzymes that regulate antioxidant gene expression [35], increasing glutathione synthesis and abundance. However, at higher doses PEITC depletes cells of glutathione through sustained intracellular conjugation and efflux [19, 20]. Glutathione depletion accompanied by compromised mitochondrial function ultimately results in excessive oxidative stress, as demonstrated by increased DNA damage with higher PEITC exposure and may explain the observed cell cycle arrest and cell cytotoxicity in MCF-7 cells treated with PEITC. Thus, the ability of PEITC to deplete glutathione availability sensitizes the MCF-7 cells to IR-induced damage resulting in G1 cell cycle arrest, greater DNA damage and reduced cell viability.

Interestingly, PEITC did not deplete MCF-10A cells of glutathione and these cells were also less sensitive to PEITC-induced DNA damage. PEITC has previously been shown to selectively kill cancer cells over non-tumorigenic cell lines due to their lower antioxidant status [36–38]. When MCF-10A cells were exposed to IR and PEITC (10 µM), glutathione was depleted as it scavenges IR-derived ROS and to compensate for this loss glutathione synthesis was up-regulated. This metabolic adaption may explain the lower DNA damage seen in MCF-10A cells compared to MCF-7 cells following IR.

Cellular membranes are a primary target of IR due to the impact ROS can have on lipid bilayers, of which phosphocholine is a main constituent. Following IR, phosphocholine was increased in the MCF-10A cells. This increase may reflect the efforts of the cell to maintain membrane integrity, which is violated by ROS produced during IR. This is consistent the greater abundance of glutathione in MCF-10A cells and their greater resistance against oxidative damage. Conversely, phosphocholine was reduced in the MCF-7 cells in response to IR. Decreases in phosphocholine have been observed in tissues after chemotherapy and radiation treatment and have been correlated with positive therapy outcomes [32, 39–41].

In both cell types, the watercress extract increased the cellular glutathione content. This is likely to be a result of the complex mixture of compounds in the watercress extract such as phenolics and flavonoids with proven antioxidant properties. Flavonoids increase the expression of γ-glutamylcysteine synthetase, which is directly proportional to glutathione abundance [42]. Watercress is also a rich source of folate [43] which can be used in one-carbon metabolism pathway, adding to the cellular glutathione pool. Several in vitro studies have shown the anti-genotoxic properties of watercress extracts [10, 44]. In the MCF-10A cells, pre-treatment with the watercress extract had a protective effect against IR exposure, evidenced by lower DNA damage compared to MCF-7 cells additional to increased glutathione content. This suggests enhanced antioxidant activity and hence a protective effect. The presence of additional antioxidant compounds in watercress help to preserve the glutathione content of the cells.

PEITC and watercress strongly interact with the metabolism of amino acids in both cell lines. PEITC at the higher doses, but not watercress, induced a strikingly selective increase in the pool of amino acids in MCF-7 cells, but not in the MCF-10A cells. These effects were generally maintained after IR. Rapidly dividing cells rely heavily on the maintenance of their biosynthetic potential as well as redox status for survival. Continuous shuttling of carbon molecules through amino acids such as glycine, methionine, threonine and serine, in the one-carbon metabolism pathway, which has a central role in cell proliferation and cancer progression, ensures the availability of the building blocks necessary for the construction of new cellular components. This also sustains the formation of reducing power compounds for redox balance. Accumulation of amino acids in the PEITC-treated MCF-7 cells is suggestive of a blockage in one-carbon metabolism pathway resulting in the inability of these cells to maintain their needs in macromolecules necessary for proliferation, increasing their susceptibility to IR damage.

Amino acids are key components for the protein translational requirements of cancer cells. Elevated mRNA translation is a key driver of carcinogenesis and PEITC has been recently shown to increase eIF2a phosphorylation and inhibit mTORC1 activity resulting in inhibition of translation in MCF-7 cells and in B cells from chronic lymphocytic leukaemia [45]. Further investigation is needed to understand if the accumulation of amino acids seen in the PEITC-treated cells is a cause or consequence of translational inhibition.

mTORC1 is master regulator of protein translation, which is a known target of PEITC [46, 47]. PEITC causes mitochondrial damage that essentially increases the AMP/ATP ratio due to energy depletion, which in turn activates AMPK. AMPK acts upstream of mTORC1, ultimately inactivating it and suppressing translation. MCF-10A cells have a lower basal mTORC1 activity as compared to the MCF-7 cells [48] suggesting that PEITC has a stronger affinity for cells with increased rates of translation.

The chemopreventive potential of watercress and its effects against oxidative stress have been investigated in a number of in vivo studies. Although pharmacokinetic data for PEITC following the ingestion of watercress is limited, Ji et al. reported a mean maximal PEITC plasma concentration (*C*_max_) of 929 nM following the consumption of 100 g of watercress [49] while Alwi et al. [50] reported a *C*_max_ of 297 nM with 80 g watercress. In the study by Alwi et al., this single 80 g of portion of watercress was sufficient to reduce the phosphorylation of 4E-binding protein 1, a key factor in angiogenesis. In another study, a single 50 g portion of watercress effectively attenuated the immunoreactivity of a proinflammatory cytokine macrophage migration inhibitory factor [51]. Daily intake of 85 g of watercress for 8 weeks has been shown to decrease DNA damage in peripheral blood lymphocytes and lipid peroxidation [8, 9] These in vivo studies demonstrate that dietary intake of watercress is sufficient to modulate anticarcinogenic pathways.

## Conclusions

These results suggest a potential synergistic effect of PEITC and IR towards MCF-7 cell killing and radiosensitization and that watercress extract, free of PEITC, can rescue healthy cells from collateral damage. It is postulated that glutathione has a principle role in the response of cells to IR challenge and that the inclusion of dietary watercress during RT may enhance the outcome. The systemic impact of watercress against breast cancer should be further explored in the clinical setting, as well as the response of the breast cell lines to IR following treatment with a combination of the watercress extract and PEITC, for the generation of robust evidence in support of the current findings.

## Electronic supplementary material

Below is the link to the electronic supplementary material.

**Fig. S1**: (a) Correlation coefficients plot obtained from the OPLS-DA model identifying metabolic changes in the MCF-7 cells induced by 5 Gy of IR exposure. (b) OPLS-DA model constructed on the metabolic profiles of cell extracts obtained from control and irradiated (5 Gy IR exposure) MCF-10A cells (c) OPLS-DA coefficients plot comparing the metabolic profiles of untreated control MCF-7 cells and the highest dose of WX (50 μl/ml) treated cells. (d) OPLS-DA coefficients plot comparing the metabolic profiles of untreated control MCF-7 cells and PEITC (20 μM) treated cells. (e) OPLS-DA coefficients plot comparing the metabolic profiles of untreated control MCF-10A cells and the highest dose of WX (50 μl/ml) treated cells. (f) OPLS-DA coefficients plot comparing the metabolic profiles of untreated control MCF-10A cells and PEITC (20 μM) treated cells. AXP: indistinguishable difference between AMP, ADP, ATP, GPC, glycerophosphocholine (DOCX 279 KB)

## Abbreviations

IR: Ionising radiation
NMR: Nuclear magnetic resonance
OPLS-DA: Orthogonal projections to latent structures-discriminant analysis
PCA: Principal component analysis
PEITC: Phenethyl isothiocyanate
*Q*^2^*Y*: Predictive ability parameter of OPLS-DA model
WX: Watercress
