# Supplementary figures and images for: Metabolic targets of watercress and PEITC in MCF-7 and MCF-10A cells explain differential sensitisation responses to ionising radiation
Source: Eur J Nutr. 2018 Jul 31;58(6):2377–91. doi: 10.1007/s00394-018-1789-8 (PMC6689287; doi:10.1007/s00394-018-1789-8)

**
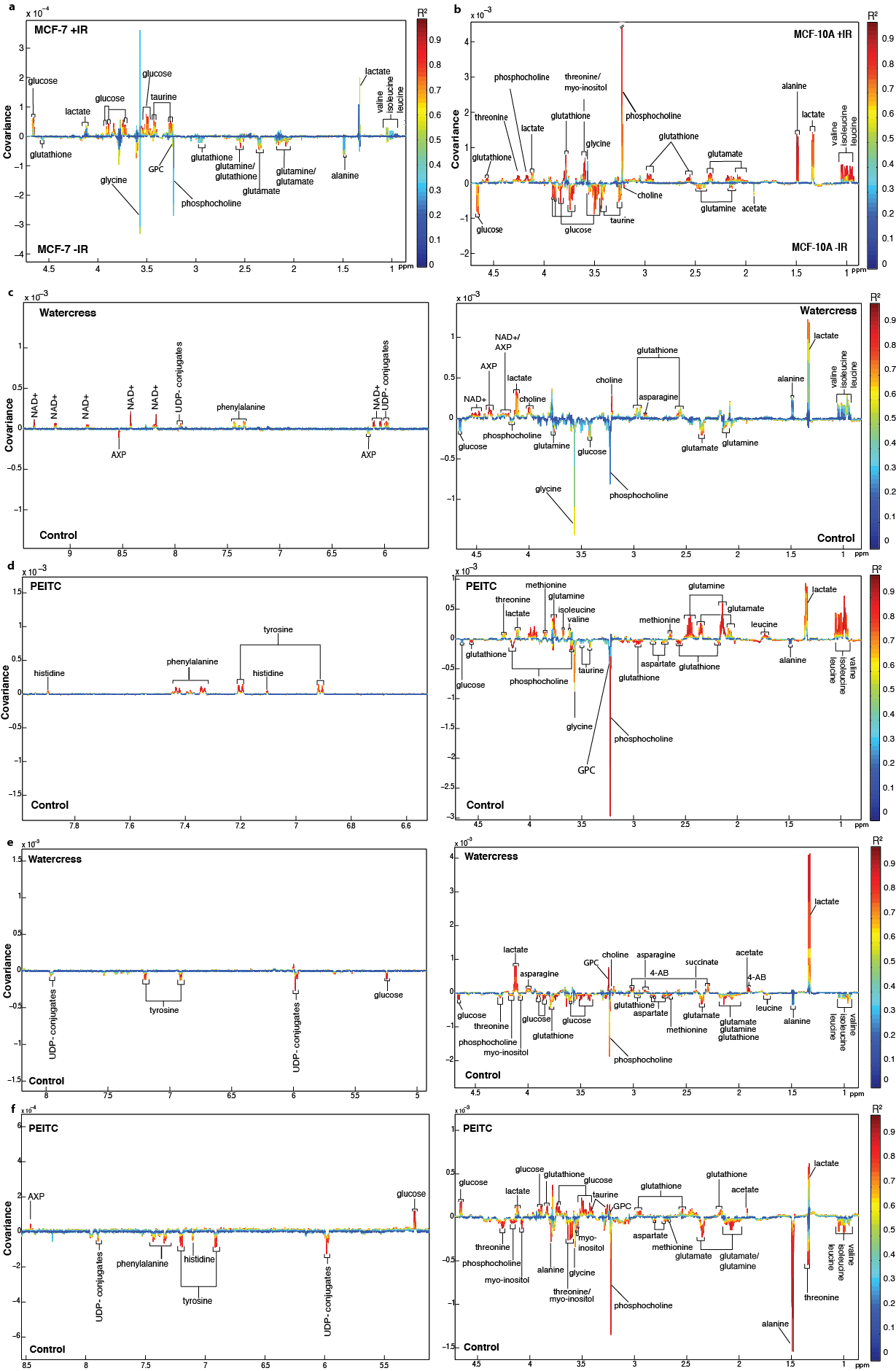
**

Figure S1

Supplement: Supplementary file 1 — Fig. S1: (a) Correlation coefficients plot obtained from the OPLS-DA model identifying metabolic changes in the MCF-7 cells induced by 5 Gy of IR exposure. (b) OPLS-DA model constructed on the metabolic profiles of cell extracts obtained from control and irradiated (5 Gy IR exposure) MCF-10A cells (c) OPLS-DA coefficients plot comparing the metabolic profiles of untreated control MCF-7 cells and the highest dose of WX (50 μl/ml) treated cells. (d) OPLS-DA coefficients plot comparing the metabolic profiles of untreated control MCF-7 cells and PEITC (20 μM) treated cells. (e) OPLS-DA coefficients plot comparing the metabolic profiles of untreated control MCF-10A cells and the highest dose of WX (50 μl/ml) treated cells. (f) OPLS-DA coefficients plot comparing the metabolic profiles of untreated control MCF-10A cells and PEITC (20 μM) treated cells. AXP: indistinguishable difference between AMP, ADP, ATP, GPC, glycerophosphocholine (DOCX 279 KB) [file 394_2018_1789_MOESM1_ESM.docx]
